# The Use of Exogenous Lung Surfactant (Poractant Alfa) in Acute Respiratory Failure by Drowning

**DOI:** 10.1155/2020/9270791

**Published:** 2020-06-06

**Authors:** Mario Pezzi, Francesco Givigliano, Ottorino Perrone, Annamaria Scozzafava, Pietro Maglio, Patrizia Casella, Anna Maria Giglio, Mario Verre, Carlo Pietro Voci

**Affiliations:** Anesthesia and Intensive Care Department, University Department of Thoracic Surgery General Hospital “Pugliese-Ciaccio”, Catanzaro, Italy

## Abstract

Drowning is an acute respiratory failure as a result from immersion or submersion of the airways in a liquid medium (predominantly water). Inhalation of water causes severe lung damage due to the destruction of pulmonary surfactant, resulting in decreased lung elasticity, alveolar collapse, alteration of ventilation-perfusion ratio, intrapulmonary blood shunting, hypoxia, acute lung injury, and Acute Respiratory Distress Syndrome (ARDS). Poractant alfa (Curosurf®), a natural surfactant effective in the treatment of newborn respiratory distress, has been used in various forms of ARDS, but in drowning syndromes, experience is still poor. We describe a series of nine clinical cases of drowning, six adults and three children, treated in our Intensive Care Unit (ICU) with endobronchial administration of poractant alfa. After 24 and 48 hours of administration in all cases, there was an improvement in arterial blood gas analysis (ABG) parameters and imaging. All patients were discharged without clinical consequences.

## 1. Introduction

Drowning is defined as “the process of experiencing respiratory impairment from submersion/immersion in liquid” [1].

In Italy, approximately 400 deaths per year occur by drowning with an average of around 6-7 deaths per million inhabitants/year. About 450 patients are hospitalized from drowning [2].

The Szpilman Classification of 1997 is a valid guide for the therapeutic strategy based on the importance of respiratory and haemodynamic disorders observed initially [3]. It also has the following prognostic values: grade 1: normal pulmonary auscultation with coughing; grade 2: abnormal pulmonary auscultation with rales in some pulmonary fields; grade 3: acute pulmonary edema without arterial hypotension; grade 4: acute pulmonary edema with arterial hypotension; grade 5: isolated respiratory arrest; and grade 6: cardiopulmonary arrest.

The sequence of drowning events begins with an unexpected dive, followed by rapid retention of breath, voluntary apnea, and panic. This apnea can last a few minutes until the “breaking point” in which the association between the inspiratory command and the cerebral hypoxia leads to the resolution of the spasm and the resumption of respiratory movement with increased inhalation. After hypoxia occurs, there is loss of consciousness, active aspiration of fluid, seizures, and death (wet drowning). In a small number of victims, severe laryngospasm causes hypoxia, seizures, and death in the absence of active aspiration (dry drowning). Hypothermia is a constant during drowning. Traumatic lesions can also be associated with underwater accidents, in particular, cervical spinal cord injuries.

Survival depends on several interrelated factors, which include water temperature, duration and degree of hypothermia, immersion reflex, age of the victim, water contamination, duration of cardiac arrest, precocity and efficacy of initial treatment, and cerebral resuscitation [4].

The consequences of the inhalation of salt water or fresh water are similar. Severe pulmonary lesions may develop in patients who survive after timely resuscitation. The contact of the liquid with the alveoli alters the surfactant, leading to an alveolar collapse with formation of atelectasis and an increase in the permeability of endothelial cells. The consequences are those of a pulmonary edema with alteration of the ventilation/perfusion ratio, increased intrapulmonary shunt, and decreased pulmonary compliance with increased respiratory work. Pulmonary lesions may evolve into ARDS. Multiple Organ Dysfunction Syndrome (MODS) can develop in patients who survive drowning [5].

## 2. Procedures

We report our experience on the use of poractant alfa (Curosurf®) administered through a bronchoscope in nine patients, six adults and three children, with acute respiratory failure by drowning.

For the off-label use of the life-saving drug, we obtained the written informed consent from the patient's authorized relatives and the authorization from the hospital's medical direction.

The patients were admitted to the ICU after the first aid performed at the accident site and were assessed according to Szpilman's Classification.

The presence of concomitant traumas has been reported.

At the time of admission in the ICU, an ABG with evaluation of the ratio Oxygen Arterial Partial Pressure/Oxygen Inspired Fraction (PaO2/FiO2) was practiced.

All patients underwent pulmonary ventilation according to the ARDS-net protocol with a low-volume, low-pressure ventilation, which targets a tidal volume of 6 ml/kg (predicted body weight), a plateau airway pressure (Pplat) of ≤30 cm H_2_O. The positive end-expiratory pressure (PEEP) and FiO2 have been adjusted in such a way as to obtain a peripheral oxygen saturation (SpO2) 88–93% or PaO2 55–80 mmHg. We chose the ventilatory mode in pressure-controlled ventilation with guaranteed volume (PRVC).

After 6 hours of protective ventilation, the index PaO2/FiO2 has been reassessed and so the poractant alfa was administered in the bronchi, by the flexible fiberoptic bronchoscope inserted through the endotracheal tube just after a thorough aspiration of secretions present in the bronchial tree.

In order to prevent hypoxia during the bronchoscopic procedure, an alveolar recruitment maneuver, which included a 40-second breath-hold at 40 cm H_2_O airway pressure, on an FiO2 of 1.0 [6], was performed before surfactant instillation. To achieve this, the CPAP (continuous positive airway pressure) mode of the ventilator is used by setting the desired pressure level.

Dilution of the drug was established to administer a total dosage of 20-30 mg/kg based on the ideal body weight of the patients. In adult patients, 1200-1440 mg of the drug diluted in 100 ml of 0.9% saline with a concentration of 12-14.4 mg/ml was used, and 20 ml of the solution (240-288 mg) was administered for each pulmonary lobe through the bronchoscope 4.8 mm. In children, 480-720 mg of the drug diluted in 50 ml of 0.9% saline with a concentration of 9.6-14.4 mg/ml was used, and 10 ml of the solution (96-144 mg) for each pulmonary lobe was administered through the pediatric bronchoscope of 3.3 mm.

We chose a low dosage (20-30 mg/kg) of ideal body weight (IBW) of the drug instilled in the bronchi based on literature data on the use of the exogenous surfactant in adults and children (Table 1). While for the treatment of newborn respiratory distress syndrome a dosage of 100 mg/kg is necessary, in a child and in an adult, a dosage of 20 mg/kg is considered effective [7–9].

In all patients, the aspiration of tracheobronchial secretions was avoided for at least thirty minutes after the instillation of poractant alfa.

During bronchoscopy and in the following fifteen minutes, a FiO2 of 1 was maintained. After fifteen minutes, FiO2 was restored to its previous value, accepting a minimum value of SpO2 > 88%. In cases where the value of SpO2 was less than 88%, FiO2 was increased to maintain this value. The PaO2/FiO2 ratio was revalued after 6, 24, and 48 hours (Table 2).

After 24 hours of endobronchial poractant alfa administration, Rx or chest TC was repeated (Table 3).

## 3. Results

### 3.1. Discussion

The ARDS, according to the Berlin definition, is classified as mild, moderate, and severe on the basis of the PaO2/FiO2 ratio value, with the minimum level of PEEP of 5 cmH2O that excludes hypoxemia caused by atelectasis: (1) mild: PaO2/FiO2 ratio greater than 200 mmHg, but less than 300 mmHg; (2) moderate: PaO2/FiO2 ratio exceeding 100 mmHg, but less than 200 mmHg; and (3) severe: ratio PaO2/FiO2 not exceeding 100 mmHg. Imaging criterion of ARDS is bilateral infiltrate on chest X-ray that cannot be explained by effusion, collapsed lung, or lung nodule. Additionally, imaging can be derived from thorax CT [10].

In the development of ARDS, a fundamental role is assumed by the alteration of the pulmonary alveolar surfactant: lack of surface-active compounds and alteration of phospholipid, fatty acid, and apoprotein profiles; altered distribution of surfactant subtypes; inhibition of surfactant function by plasma protein leakage; incorporation of surfactant in fibrin/hyaline membranes; and damage of surfactant compounds by inflammatory mediators [11].

Poractant alfa is a lung surfactant prepared from minced porcine lungs and is useful for the treatment of neonatal respiratory distress syndrome. Clinical formulations contain 1% hydrophobic proteins (surfactant proteins B and C). Beneficial effects of poractant alfa are related to its ability to lower surface tension in the lung, increase lung compliance, and prevent atelectasis at end expiration via a stabilizing effect on the alveoli [12]. Exogenous surfactants have been used as complementary therapy in the treatment of ARDS in many clinical trials, reporting in all a benefit in the pulmonary function measured through the improvement of oxygenation, with no significant reduction in mortality, nor for the time of ventilation, nor the ICU stay [7] [13]. Positive results have been reported in children treated with endobronchial surfactant [14–16].

An Italian multicenter study showed improvements in oxygenation, PaO2/FiO2 ratio, oxygenation index, and pH in a population of children with moderate or severe ARDS caused by multiple diseases [17]. Another multicenter study conducted in Cuba on children with ARDS showed improvements in respiratory parameters and survival [18].

The use of selective bronchoalveolar lavage with saline+natural surfactant has shown facilitation in the removal of degraded lung material and in recruiting the contused lung regions in a randomized study in patients with traumatic pulmonary contusions [8].

In drowning syndromes with acute respiratory failure, the use of exogenous surfactant has been reported in a few cases of adult patients [19] and several cases of pediatric patients [20–25]. All reported cases had a positive outcome, but there are no double-blind controlled multicenter studies to demonstrate the superiority of surfactant treatment compared to traditional treatment.

## 4. Conclusions

In all patients treated by us with endobronchial poractant alfa, an improvement of the PaO2/FiO2 ratio and radiological data was shown after 24 and 48 hours. The healing process continued over the following days without complications.

Our case series is too small to provide certainty about the efficacy of endobronchial administration of poractant alfa in drowning syndromes. To confirm the usefulness and effectiveness of the exogenous surfactant in drowning syndromes, controlled polycentric studies would be needed.

## Figures and Tables

**Table 1:** Patients' characteristics and instilled dose.

Patient	1	2	3	4	5	6	7	8	9
Gender	Male	Male	Female	Male	Male	Male	Male	Female	Female
Age (yrs)	78	76	6	78	9	18	76	45	10
Height (cm)	170	172	115	173	125	180	182	150	135
Weight (kg)	82	78	21	84	30	70	85	65	38
Ideal body weight	65.9	67.7	23.5	68.7	31.1	75	76.8	47.3	37.5
Instilled dose of surfactant (mg/kg)	21.9	21.3	22.5	20.9	23.7	20.2	18.75	25.36	22.5
Concurrent lesions	None	Head trauma fracture of the skull by a motorboat propeller	None	None	None	Blunt head and cervical spine trauma by a dive	None	None	None

**Table 2:** Classification and evolution of the PaO2/FiO2 ratio.

Patient number	Grade of Szpilman Classification	PaO2/FiO2 at the time of admission to the ICU	PaO2/FiO2 immediately before drug administration	PaO2/FiO2 after 6 hours	PaO2/FiO2 after 24 hours	PaO2/FiO2 after 48 hours
1	Grade 6	101	108	134	179	206
2	Grade 4	97	102	127	185	210
3	Grade 5	115	121	158	212	268
4	Grade 6	96	98	115	138	175
5	Grade 4	114	119	173	232	412
6	Grade 2	200	220	288	310	407
7	Grade 5	107	122	179	212	315
8	Grade 3	121	143	161	198	274
9	Grade 5	112	128	179	255	367

**Table 3:** Imaging.

Patient number and kind of inhaled water	Rx or CT of the thorax at the time of administration of the drug	Rx or CT of the thorax after 24 hours
No. 1 Fresh water	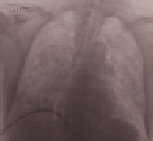	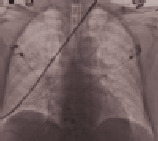
No. 2 Seawater	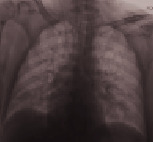	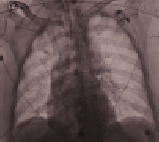
No. 3 Seawater	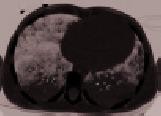	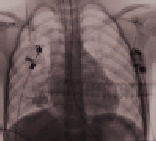
No. 4 Fresh water	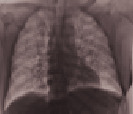	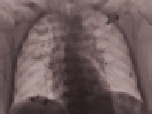
No. 5 Seawater	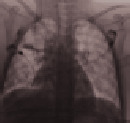	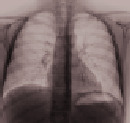
No. 6 Seawater	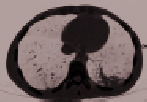	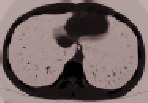
No. 7 Seawater	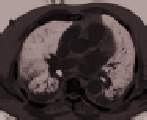	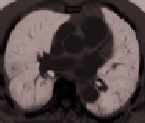
No. 8 Seawater	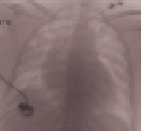	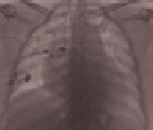
No. 9 Fresh water	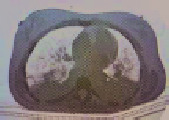	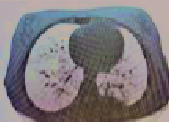

## Data Availability

The authors confirm that the data supporting the findings of this study are available within the article.
